# Influences of Gastrointestinal Microbiota Dysbiosis on Serum Proinflammatory Markers in Epithelial Ovarian Cancer Development and Progression

**DOI:** 10.3390/cancers14123022

**Published:** 2022-06-20

**Authors:** Diane E. Mahoney, Prabhakar Chalise, Faith Rahman, Janet D. Pierce

**Affiliations:** 1 School of Nursing, University of Kansas Medical Center, Kansas City, KS 66160, USA; jpierce@kumc.edu; 2 Department of Biostatistics and Data Science, University of Kansas Medical Center, Kansas City, KS 66160, USA; pchalise@kumc.edu; 3 Clinical Trials Clinical Operations, University of Kansas Cancer Center, Kansas City, KS 66160, USA; frahman2@kumc.edu

**Keywords:** epithelial ovarian cancer, gastrointestinal microbiota dysbiosis, proinflammatory markers

## Abstract

**Simple Summary:**

Advanced epithelial ovarian cancer (EOC) is the deadliest gynecologic cancer due to the lack of available early screening methods that are available in clinical settings. Unique gastrointestinal (GI) microbiota have been detected in the ovaries and peritoneal fluid of women with EOC that are not found in women without disease. However, there are limited data available to determine possible links in GI microbiota composition and early ovarian cancer development while women are largely asymptomatic. This research utilizes an EOC animal model to investigate the effects of GI microbial disruption on systemic inflammation during early disease development and progression. The identification of distinct GI microbial communities that may potentially mediate systemic immune response during carcinogenesis can open new opportunities to disentangle the complex ovarian tumor microenvironment for the discovery of valid clinical tools for early EOC detection.

**Abstract:**

GI microbiota has been implicated in producing the inflammatory tumor microenvironment of several cancers. Women with ovarian cancer often report GI-related symptoms at diagnosis although minimal is known about the possible GI bacteria that may trigger pro-tumorigenic immune responses in early EOC. The purpose of this study was to investigate the influences of GI microbiota dysbiosis on serum inflammatory markers during EOC utilizing a rodent model. This experimental design consisted of C57BL/6 mice randomly assigned to either the microbiota dysbiosis group (*n* = 6) or control group (*n* = 5). The CD7BL/6 mice assigned to the microbiota dysbiosis group were administered a mixture of broad-spectrum antibiotics (bacitracin and neomycin) for 2 weeks. Both groups were injected intraperitoneally with mouse ovarian epithelial cells that induce ovarian tumorigenesis. Levels of C-reactive protein (CRP), interleukin-6 (IL-6), and tumor necrosis factor-alpha (TNF-α) were assessed in the serum, and the composition of the GI microbiota in fecal samples was measured using 16S rRNA gene sequencing. Overall CRP serum levels were significantly lower and TNFα levels were significantly higher in the microbiota dysbiosis group compared to the control group. The abundances of microbiota that correlated with CRP serum levels in the combined groups were genus Parabacteroides, Roseburia, and Emergencia and species Ruminococcus faecis, Parabacteroides distasonis, Roseburia Faecis, and Emergencia timonensis. This study provides evidence to support for further investigation of the GI microbial profiles in patients at risk of EOC.

## 1. Introduction

Despite the development of improved therapies to overcome disease, ovarian cancer remains the deadliest cancer of the female reproductive tract [1,2]. In 2022, an estimated 19,880 women in the U.S. will be diagnosed with ovarian cancer, and nearly 13,000 women will die of the disease [3]. Ninety percent of ovarian cancers are epithelial, and the majority (60%) are high-grade serous tumors that metastasize into the peritoneal cavity [1,4,5]. The remaining 10% of ovarian cancers are of nonepithelial histology, which includes sex cord stromal and germ cell tumors. Malignant ovarian carcinosarcoma is a rare type accounting for 1–4% of all ovarian cancers with unknown histogenesis [6,7]. Advanced epithelial ovarian cancer (EOC) is associated with an overall 30% survival rate, but it can be cured in up to 90% of cases if diagnosed while still limited to the ovaries and fallopian tubes [3,8]. Unfortunately, most women with EOC are asymptomatic in the initial stage of disease and remain undiagnosed until the disease reaches advanced stages, resulting in overall poor survival rates [9,10]. Women commonly report GI-related symptoms at the time of diagnosis as ovarian tumors become more distant and metastasize. Thus, noninvasive clinical tests are needed to identify early disease in women, particularly women at higher risk. In pursuit of biomarkers of early disease detection, the field of proteomics has demonstrated the important clinical relevance of ovarian cancer diagnostics because the proteome closely mimics the dynamic state of human cells, tissues, and organs [11]. Furthermore, advanced proteomics technologies have helped drive our understanding of the human microbiome [12]. Thus, researchers are increasingly focused on human microbiota, the collection of microorganisms that live in the body, as potential disease biomarkers [13]. Specifically, gastrointestinal (GI) microbiota have emerged as significant influencers of health and disease within the gut and beyond to other organs [14]. Furthermore, GI microbiota are implicated in shaping the inflammatory tumor microenvironment in GI cancers, although the specific role of GI microbiota in ovarian cancer is underexplored [15,16]. A crucial characteristic of ovarian cancer biology is peritoneal diffusion, and the unique profiles of bacterial phyla (Proteobacteria, Firmicutes, Acinetobacter, and Lactococcus) have been detected in the peritoneal fluid of women with EOC [9,17,18,19]. However, scientists have not determined if EOC-associated bacteria can be detected elsewhere in the body sites during ovarian carcinogenesis when women are asymptomatic. There is minimal knowledge about specific bacteria that trigger pro-tumorigenic immune responses in early disease.

Microorganisms have been shown to modulate known human host factors that comprise the hallmarks of cancer [20]. A fundamental concept in ovarian tumorigenesis is chronic inflammation in the tumor microenvironment and systemically although, the precise immunologic mechanisms are unclear [21,22]. Evidence suggests that chronic inflammation creates a pro-tumorigenic environment through the production of proinflammatory markers, angiogenesis, and tissue remodeling [23]. Proinflammatory–microbiota interactions have revealed cytokine- and stimulus-related patterns that influence the immune response [24]. It is plausible that GI microbiota and their toxic metabolites could migrate to other regions of the body via intricate pathways that promote chronic inflammation and tumorigenesis [25]. In addition to peritoneal fluid, GI tract (commensal) bacteria have been detected in the ovarian tumors of women with EOC that differ from those that found in women with benign gynecologic conditions [26]. Preclinical models are proposed to understand possible mechanistic functions that may interlink these microorganisms with local and systemic inflammation during EOC development. C-reactive protein (CRP), interleukin-6 (IL-6), and tumor necrosis factor-alpha (TNF-α) serum markers have shown to be higher in women with malignant ovarian tumors while there is uncertainty if serum levels are associated with unique GI microbes [27,28,29,30]. Thus, the purpose of this research was to explore the influences of GI microbiota dysbiosis on inflammatory markers during EOC development and progression using a mouse model. We postulated that serum levels of CRP, IL-6, and TNF-α would increase in response to compositional alterations in GI microbiota during ovarian carcinogenesis. 

## 2. Materials and Methods

### 2.1. C57BL/6 Mice

All study procedures were reviewed and approved by the University of Kansas Medical Center Animal Care and Use Committee (Protocol Registry Number: 2020-2576). Female 8-week-old C57BL/6 mice were obtained from Jackson Laboratories (Bar Harbor, ME, USA) and housed in the university experimental animal facility. Procedures for treatment and care of the mice were followed in accordance with the institutional guidelines.

### 2.2. ID8 Cells

**Figure 1:**
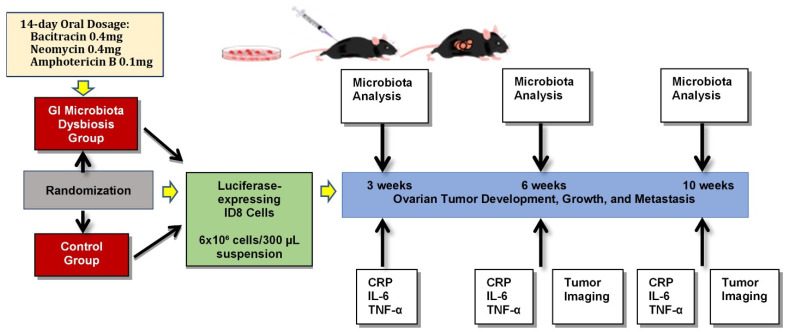
Experimental design.

ID8 cells (provided by Dr. Katherine F. Roby, Anatomy and Cell Biology, University of Kansas Medical Center, Kansas City, KS, USA) were transfected with an mCherry/luciferase-encoding lentiviral system to express mCherry and firefly luciferase [31,32]. The cells were cultured in Dulbecco’s Modified Eagle’s Medium (DMEM) containing 4% fetal bovine serum (FBS) supplemented with 100 U/mL penicillin, 100 μg/mL streptomycin, and 5 μg/mL insulin at 37 °C in a humidified 5% carbon dioxide (CO_2_) incubator. After overnight culture to allow cellular attachment to the plates, the medium was removed and fresh medium without FBS was added to prevent the effects of serum. The ID8 cells were transduced at a multiplicity of infection of 20 and subsequently selected with puromycin (5 mg/mL). ID8 cells were expanded for injection. C57BL/6 mice were injected in the intraperitoneal (IP) cavity with 6 × 10^6^ luciferase-expressing ID8 cells in 300 μL DMEM. Tumor cell growth and distribution were assessed from bioluminescence signaling of the ID8 cells using an in vivo imaging system (IVIS). A graphical representation of the study design is presented in Figure 1.

### 2.3. Epithelial Ovarian Cancer Mouse Model

The syngeneic mouse model was developed in 6- to 8-week female C57BL/6 mice by IP injection of mouse ovarian surface epithelial cells (from the ID8 clonal line) that produce tumors within the peritoneum as observed in women with Stages III and IV cancer [33]. These tumors result in ascites formation and metastasis to the diaphragm, intestinal walls, peritoneal lining, and omentum, reaching end-stage disease at approximately 90 days post ID8 cell injection [34]. The syngeneic mouse model is frequently cited in preclinical studies [35,36,37,38,39].

### 2.4. GI Microbiota Dysbiosis Model

Microbiota dysbiosis was established based on an antibiotic-induced dysbiosis mouse model [40]. The purpose of the antibiotic therapy was to alter endogenous gut bacteria and study the influence of microbiota imbalances on the inflammatory response. Animals were randomly assigned, with six mice in the GI microbiota dysbiosis (treatment) group and five in the control group. Mice in the treatment group received a mixture of broad-spectrum antibiotics (bacitracin and neomycin), and amphotericin B was also added to prevent yeast overgrowth. During a 14-day period, treatment group mice were provided 80 mL of drinking water with 10% sucrose (prepared fresh daily) to ensure a minimum dose of 0.4 mg of bacitracin (MilliporeSigma, Burlington, MA, USA) and neomycin (Wedgewood Pharmacy, Swedesboro, NJ, USA), and 0.1 mg of amphotericin B (Wedgewood Pharmacy) per mouse daily. Control group mice also received the same volume of drinking water prepared with 10% sucrose, but without the addition of antibiotics. Water consumption was measured daily and no differences between the GI microbiota dysbiosis and control groups were found during the antibiotic treatment period. After the antibiotic therapy subsided, the animals were supplied standard drinking water in accordance with the institutional animal housing facility practices. Upon completion of antibiotics, all mice were injected with the ID8 cells and monitored.

### 2.5. Flow Cytometry

Peripheral blood samples were collected at 3 weeks, 6 weeks, and 10 weeks post ID8 cell injection from the facial submandibular veins of mice using animal lancets (Braintree Scientific Goldenrod, 5 mm, Braintree, MA, USA), and placed into a microtainer capillary blood collector. Sera were collected and frozen in a −80 °C freezer and analyzed in batches. Mouse CRP concentrations were determined using 1-plex kits (MilliporeSigma, Burlington, MA, USA) under standard procedures and analyzed using the Luminex^®^ 200 instrument (University of Kansas Medical Center Flow Cytometry Core). Mouse IL-6 and TNF-α concentrations were determined using the 2-flex set system kits (MilliporeSigma) and analyzed using the Luminex^®^ 200 instrument. A standard curve was generated each time assays were run. Two assays were performed for each sample based on the recommended dilutions for serum levels according to standard procedures.

### 2.6. Bioluminescence Signal Quantification

The bioluminescence analysis was performed at 6- and 10-weeks post injection of the luciferase-expressing ID8 cells in each mouse. The study animals received IP injection of 100 μL D-Luciferin (150 mg/kg body weight in normal saline; GoldBio, St. Louis, MO, USA) to generate bioluminescence signals. After 10 min, the animals were anesthetized (PerkinElmer XGI-8 Gas Anesthesia System, Waltham, MA, USA) with isoflurane (3% vaporized in O_2_), and the bioluminescence signals were captured and analyzed using the IVIS Spectrum (PerkinElmer). The total flux of bioluminescence signaling was measured from a fixed region-of-interest (ROI) over the abdomen of the mice using the Living Image 4.0 software, which provided the total flux of photon radiance (photons/second from the surface) in each pixel within the ROI area. This is displayed as the average radiance, which is the sum of the radiance from each pixel inside the ROI divided by the number of pixels (photons/sec/cm^2^).

### 2.7. Fecal Collection and Microbial DNA Extraction

The animals were placed in temporary metabolic caging for a 12 h period for fecal sample collection at three time points (3 weeks, 6 weeks, and 10 weeks) to allow for separation of feces from urine. The metabolic caging also provided a means for collecting fecal pellet samples from individual mice. Food and water were not restricted during this time. The fecal samples were collected, stored, and frozen at −80 °C. In an effort to avoid significant variation to the raw count data, the fecal samples were processed in one batch (Charles River Laboratory, Wilmington, MA, USA). Deoxyribonucleic acid (DNA) was isolated from fecal samples using an optimized magnetic purification kit per the manufacturer’s protocol (ThermoFisher, Waltham, MA, USA). Recovery yield and DNA quality were determined by fluorometric analysis (Qubit, ThermoFisher). After 10 weeks post ID8 cell injection, the animals were euthanized.

### 2.8. Fecal Microbial 16S rRNA Gene Amplification and Sequencing

DNA concentration was adjusted to specifications and amplified using broadly reactive 16S rRNA primers spanning the V3 and V4 regions. Resulting amplified polymerase chain reaction (PCR) products were analyzed for quantity and correct product size (Bioanalyzer), and then purified and amplified with primers containing unique sample nucleotide barcodes (Illumina, San Diego, CA, USA). PCR products’ quality and quantity were further analyzed by SYBR green qPCR (Kapa Biosystems, Wilmington, MA, USA). All samples were pooled and adjusted to a normalized concentration. The DNA library pool was denatured with sodium hydroxide, normalized to optimal loading concentration, and combined with PhiX control (Illumina). Extended read lengths to 2 × 300 bp were used for cluster generation and sequencing (Illumina MiSeq). Sequences were clustered into operational taxonomic units (OTUs) at a 97% similarity cutoff, and the relative abundance was calculated for the OTUs in each fecal sample. Following the sequencing run, the sequence data were de-multiplexed based on the nucleotide barcode and compared to the One Codex Targeted Loci Database for taxonomic identification and analysis.

### 2.9. Diversity Indices

A diversity index, the variety and abundance of species in a defined unit of study, provides a quantitative measure that is often used to describe the complexity of the microbiota community [41]. A community that is dominated by only a few microbiota species is considered less diverse and might be associated with the outcome of a disease. Two common types of diversity indices, α-diversity (within samples) and beta (β)-diversity (between samples), are often studied. Alpha diversity, representing the ecological diversity, is measured by Shannon and Simpson index calculations. The Shannon index is a measure of the richness and evenness of all taxa in the samples. The Simpson index is an estimator of the evenness of the microbial abundances in the samples. Beta diversity is the relative distance or dissimilarity of microbial abundance proportions between the samples. Bray–Curtis dissimilarity distance, a measure of β-diversity, measures the quantity of differences in species populations between the samples.

### 2.10. Statistical Analysis

Differences in CRP, IL-6, and TNF-α serum levels over time between the treatment and control groups were assessed using linear mixed models for repeated-measures data using SAS procedure GLIMMIX. Similarly, changes in the Shannon and Simpson diversity indices between the groups were assessed longitudinally. Such models allow us to assess the differences in the values between the groups at each time point and the rate of change over time between the groups (i.e., interaction effect). Differences in the bioluminescent signal strength between the groups were assessed using Wilcoxon rank sum test. A similar test was used to compare the rates of change in the bioluminescent signals over time between the groups. Analyses of microbiota data were carried out at the phylum, genus, and species level. The microbiota counts at three time points (3, 6, and 10 weeks) were averaged out for each microbiota and compared their relative abundances between the groups. Relative abundance plots were made for a visual comparison of the microbiota composition between the treatment and control groups. Beta diversity indices were assessed by calculating Bray–Curtis dissimilarity distance and then creating a hierarchical dendrogram and heatmap for visualization. Association of the abundance of each microbiota with the CRP, IL-6, and TNF-α serum levels was assessed using Spearman’s rank correlation analysis. Differential abundances of microbiota between the treatment and control groups were assessed using Analysis of Compositions of Microbiomes with Bias Correction (ANCOM-BC) [42]. ANCOM-BC utilizes a linear regression framework to compare the relative abundances of the compositional nature of the microbiota data with library size adjustments and sample-specific offset terms for bias correction. Multiple testing adjustments were performed using Benjamini and Hochberg’s FDR method. Tests were considered statistically significant at a *p*-value of <0.05. All statistical analyses of the data were completed using SAS and R software.

## 3. Results

### 3.1. Bacterial Diversity of GI Microbiota Dysbiosis Mice versus Control Mice

**Figure 2:**
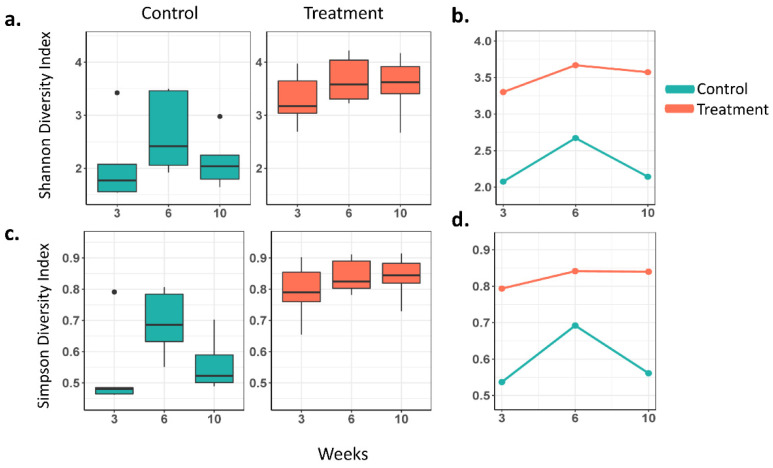
Distribution of Shannon and Simpson diversity indices in the GI microbiota dysbiosis (*n* = 6) and Control (*n* = 5) groups. (**a**) Boxplot showing the Shannon diversity index at 3, 6, and 10 weeks, (**b**) mean Shannon index over time by group, (**c**) boxplot showing the Simpson diversity index at 3, 6, and 10 weeks, and (**d**) mean Simpson index over time by groups.

To examine gut bacteria composition in the GI microbiota dysbiosis and control groups, deep sequencing of the V3 to V4 16S rRNA region was performed on all mouse fecal samples. The α-diversity of the microbes was assessed at the 3-, 6-, and 10-week time points and compared between and within each study group. Bacterial diversity was statistically significantly different between the GI microbiota dysbiosis and control mice groups (Shannon index, *p* = 0.002; Simpson index, *p* < 0.001) overall. The indices were also significantly different between the groups at each study time point (adjusted *p*-value < 0.01 at each time point for each of the Shannon and Simpson indices) (Figure 2). The rates of change of the indices over time between the groups (interaction effect) were not statistically significant.

**Figure 3:**
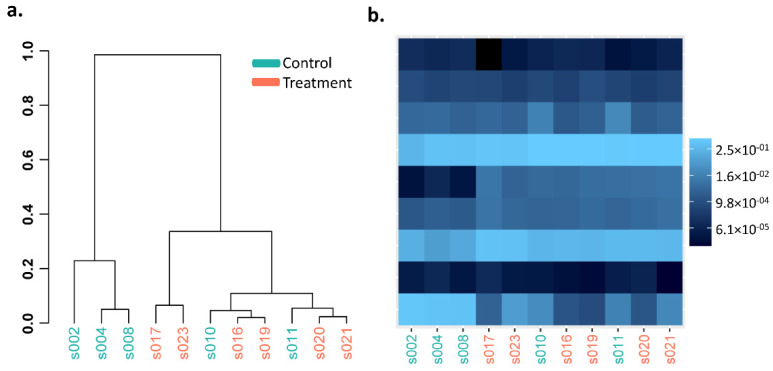
Phylum level Bray–Curtis dissimilarity comparisons in the GI microbiota dysbiosis (treatment) (*n* = 6) and control (*n* = 5) groups. (**a**) Hierarchical clustering, (**b**) heatmap.

**Figure 4:**
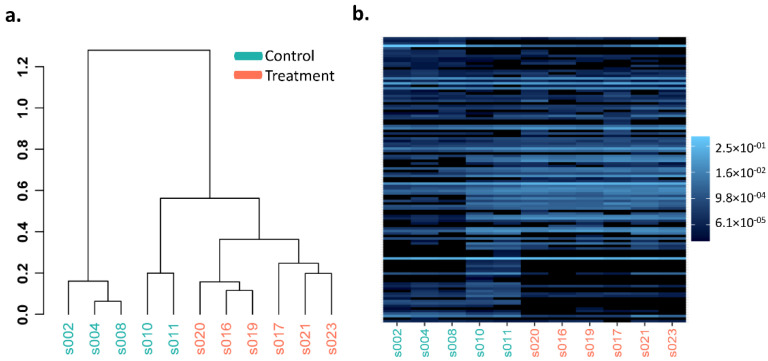
Genus level Bray–Curtis dissimilarity comparisons in the GI microbiota dysbiosis (treatment) (*n* = 6) and control (*n* = 5) groups. (**a**) Hierarchical clustering, (**b**) heatmap.

**Figure 5:**
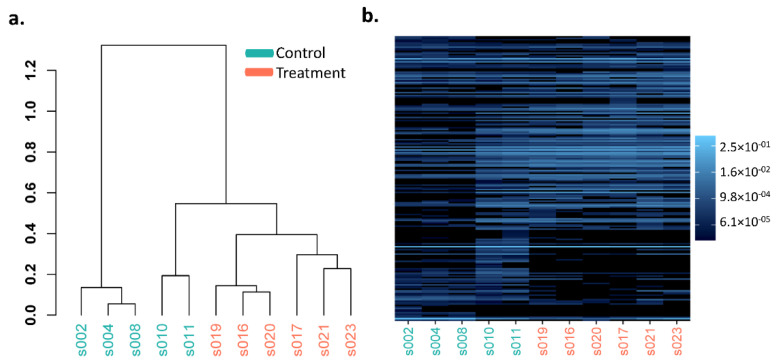
Species level Bray–Curtis dissimilarity comparisons in the GI microbiota dysbiosis (treatment) (*n* = 6) and control (*n* = 5) groups. (**a**) Hierarchical clustering, (**b**) heatmap.

**Figure 6:**
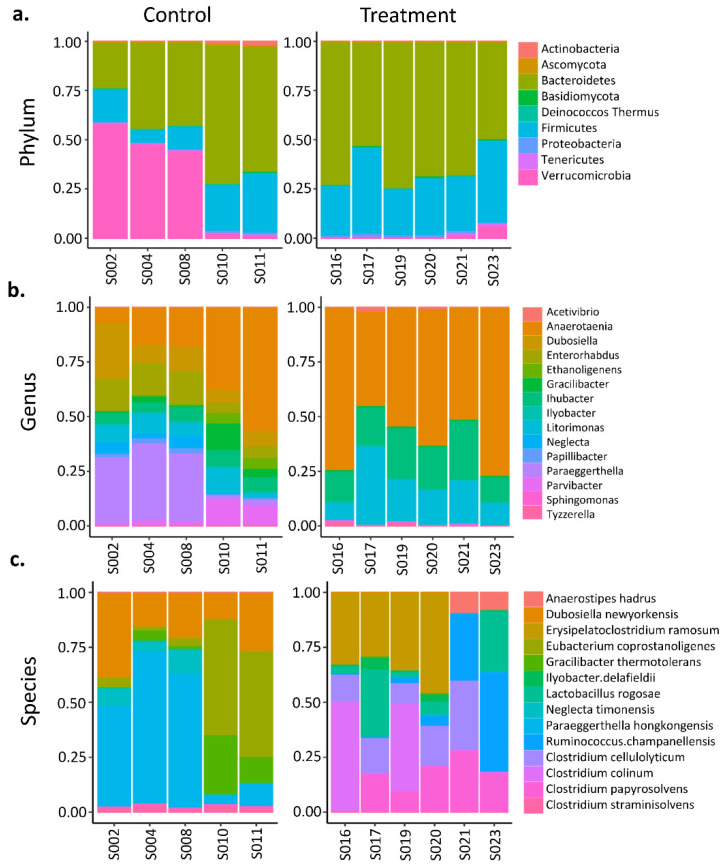
Relative abundance comparisons in the GI microbiota between dysbiosis (treatment) (*n* = 6) and control (*n* = 5) groups (**a**) at the phylum level, (**b**) gene level, (**c**) and species level.

Heatmaps and hierarchical dendrogram clusters of the Bray–Curtis dissimilarity between GI microbiota dysbiosis and control groups when clustered over the 3-,6-, and 10-week time points are shown in Figure 3, Figure 4 and Figure 5 at the phylum, genus, and species taxonomic levels. The plots indicate the differing patterns of β-diversity between treatment and control groups. Compared to the control group, the phyla Bacteroidetes, Firmicutes, Protobacteria, and Tenericutes were significantly more abundant in the GI microbiota dysbiosis group, while Actinobacteria and Verrucomicrobia were significantly less abundant (Figure 6a). At the genus level, Sphingomonas, Ilyobacter, Tyzzerella, Acetivibrio, Ihubacter, Litorimonas, Anaerotaenia were significantly more abundant in the GI microbiota dysbiosis group, while Dubosiella, Paraeggerthella, Enterorhabdus, Gracilibacter, Neglecta, Parvibacter, Ethanoligenens, and Papillibacter were significantly less abundant in the GI microbiota dysbiosis group (Figure 6b). At the species level, Clostridium cellulolyticum, Clostridium papyrosolvens, Anaerostipes hadrus, Ruminococcus.champanellensis, Erysipelatoclostridium ramosum, Lactobacillus rogosae, Ilyobacter delafieldii, and Clostridium colinum were significantly more abundant in the GI microbiota dysbiosis group, while Dubosiella newyorkensis, Eubacterium coprostanoligenes, Paraeggerthella hongkongensis, Clostridium straminisolvens, Gracilibacter thermotolerans, and Neglecta timonensis were significantly less abundant (Figure 6c).

### 3.2. CRP, IL-6, and TNF-α Serum Levels in GI Microbiota Dysbiosis Mice versus Control Mice

**Figure 7:**
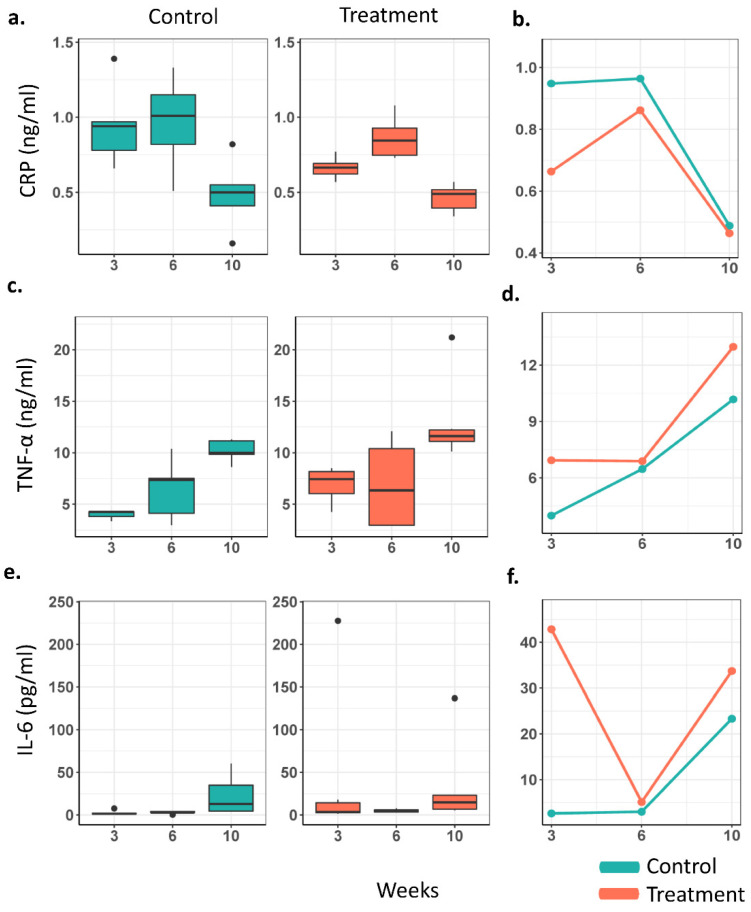
Proinflammatory serum level distribution and mean values over time in the GI microbiota dysbiosis (treatment) (*n* = 6) and control (*n* = 5) groups at 3, 6, and 10 weeks. (**a**,**b**) C-reactive protein, (**c**,**d**) tumor necrosis factor-alpha, (**e**,**f**) interleukin-6.

The relative serum levels of CRP, IL-6, and TNF-α were assayed from peripheral blood samples from the GI microbiota dysbiosis and control group at the 3-, 6-, and 10-week time points (Figure 7). CRP serum levels were significantly lower in the GI microbiota dysbiosis compared to the control group overall (*p* = 0.027) and at the 3-week time point (*p* = 0.030). In contrast, TNF-α serum levels were significantly higher in the GI microbiota dysbiosis group compared to the control group overall (*p* = 0.023). However, individual time points were not significantly different between the groups. Although there was no statistically significant difference in the rate of change in the serum levels between the groups over time (interaction), there was significant change in the serum levels within treatment or control groups separately at a few pairwise time points. For example, changes in CRP levels between the 6- and 10-week time points were significantly different in each of the treatment and control groups, and TNF-α levels significantly increased from time point 6 weeks to 10 weeks only in the treatment group. Changes were not significantly different for IL-6 levels overall or within and between the groups over time.

**Figure 8:**
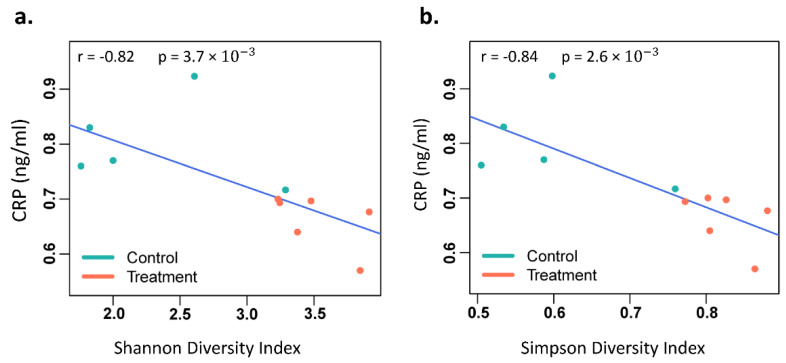
Correlation between C-reactive protein (CRP) serum level and bacterial diversity indices. (**a**) CRP and Shannon diversity index, (**b**) CRP and Simpson diversity index.

CRP, IL-6, and TNF-α serum levels and bacterial diversity composition were assessed in GI microbiota dysbiosis mice and control mice to determine possible correlations. CRP serum levels were statistically significantly correlated with the Simpson index (r = −0.84, *p* = 0.0026) and the Shannon index (r = −0.82, *p* = 0.0037) (Figure 8). IL-6 and TNF-α levels were not correlated with the α-diversity indices. At the genus level, Parabacteroides, Roseburia, and Emergencia abundance was correlated (r = 0.89, −0.83, −0.84; *p* = 0.035, 0.047, 0.044) with CRP serum levels. At the species level, Ruminococcus faecis, Parabacteroides distasonis, Roseburia Faecis, and Emergencia timonensis abundance was correlated (r = −0.83, 0.89, −0.84, −0.84; *p* = 0.047, 0.047, 0.047, 0.047) with CRP serum levels. A description of the established clinical associations of these bacteria is presented in Table 1. Details of the correlation analysis of all bacteria can be found in the Supplementary Materials (Tables S1–S9).

### 3.3. Ovarian Tumor Bioluminescence Signals in GI Microbiota Dysbiosis Mice versus Control Mice

IVIS was used to assess ovarian tumor growth at 6- and 10-weeks post ID8 cell inoculation. No significant differences were observed in the total flux of ovarian tumor bioluminescence signaling between the GI microbiota dysbiosis mice compared to the control group. Additionally, there were no differences in bioluminescence signaling measurements within the groups.

## 4. Discussion

In this study we sought to understand GI microbiota dysbiosis influences on inflammatory markers during EOC development. The mean CRP serum levels across time points were lower while TNF-α levels were higher in the GI microbiota dysbiosis mice compared to control mice. However, no differences were observed within the study time points between the two groups. Nonetheless, increased serum levels of CRP and TNF-α have been detected in women as early as 5 years prior to the time of ovarian cancer diagnosis, although there is uncertainty as to whether these markers are reflective of local site inflammation pertinent to the ovarian tumor microenvironment [56]. Proinflammatory–microbiota interactions have revealed cytokine- and stimulus-related patterns that the influence immune response [24]. It is plausible that GI microbiota and their toxic metabolites could relocate to other regions of the body via intricate pathways that promote chronic inflammation and tumorigenesis [25]. Miao and colleagues suggest GI bacterial migration as a possible mechanism to explain the presence of commensal gut bacteria detected in the peritoneal fluid of women with ovarian cancer [17]. Others suggest that local bacterial expansion within the peritoneum may facilitate the formation of metastatic disease [57]. Furthermore, elevated serum and peritoneal fluid IL-6 concentrations have been reported in women with early- and late-stage EOC compared to those with benign gynecologic conditions [58]. Moreover, in other cancers, elevated IL-6 levels have been linked to treatment resistance and reduced survival [59]. Although no differences were found in IL-6 serum levels between groups in this study, additional investigation is warranted in patients with ovarian cancer.

While differences in bacterial diversity were observed between the GI microbiota dysbiosis and control mice, these differences did not influence ovarian tumor activity in this study. These findings are inconsistent with prior research in which the ovarian tumors of GI dysbiosis mice were considerably larger than those of controls [60]. Furthermore, in this study, the same bacteria that correlated with IL-6, CRP, and TNF-α levels in the GI microbiota dysbiosis mice have been detected in fecal and tumor samples of patients with breast, cervical, and colorectal cancer [45,61,62]. Although the microbial composition of the ovarian tumor tissue was not assessed in the current study, recent research findings have shown unique noninfectious intratumor bacteria in women with ovarian cancer. Nonetheless, it is uncertain how these microorganisms impact ovarian tumorigenesis and metastasis. This suggests that bacterial metabolites (i.e., free fatty acids, amino acids) could influence immune response throughout the disease process. While the GI microbiota taxonomic annotation is specific to humans and mice, Bacteroidetes and Firmicutes are the most dominant phyla in both [63]. In the current study, Bacteroidetes, Firmicutes, and Protobacteria abundance was higher in the GI dysbiosis mice. These bacteria are also observed in the ovaries, fallopian tubes, and peritoneal fluid of women with ovarian cancer [26]. Thus, crucial factors affecting the GI microbiota, such as diet and nutrition, may have a role in ovarian cancer including treatment response. Short-chain fatty acids are the primary metabolites produced by the GI microbial fermentation of dietary fiber [64]. Acetic and propionic acids are short-chain fatty acid metabolites of Bacteroidetes, and butyric acid is a short-chain fatty acid metabolite of Firmicutes. Both bacteria are abundant in the human colon [64,65]. Bacteria colonization within the ovarian tumor microenvironment could be associated with GI-related functional molecular pathways involving bacterial metabolites. Rigorous evaluation of GI microbial composition and function could provide biomarkers of ovarian carcinogenesis.

Differences were identified in the microbial abundances between the GI dysbiosis and control groups at the phylum, genus and species levels, and some bacteria correlated with CRP serum levels. Moreover, unique to this preclinical study, is the identification of distinct bacteria in the combined study groups that have been detected in patients from other cancer populations. Bacteria biosignatures could provide a systematic roadmap to the development of microbial-based ovarian cancer screening tests used in routine care for women. Biomarker discovery research in this field has been largely limited to retrospective analyses rather than prospective investigations to compare women with ovarian cancer to those with benign conditions and to controls. Thus, the clinical applicability of these bacteria warrants additional investigation in patients at high risk of EOC (i.e., BRCA1/2 gene mutation carriers) in the absence of clinical signs and symptoms. For example, women with ovarian cancer have fewer vaginal Lactobacillus species compared to controls. Specifically, the vaginal communities of younger women with BRCA1/2 gene mutations are Lactobacillus species-poor, suggesting a role of vaginal dysbiosis in women at higher risk of EOC development [66].

One study limitation was that the bacterial diversity detected between the two study groups did not seem to influence ovarian tumor growth measured by the bioluminescence IVIS in C57BL/6 mice. Black mice have substantial lower bioluminescent signals as compared to white, shaved, or nude mice [67]. Therefore, shaving the mice could have facilitated optimal tumor growth assessment when using IVIS. Additionally, postmortem extraction of the tumors for weight measurement would have been another reasonable alternative. Another limitation is the uncertainty of the preexisting GI bacterial diversity profiles of mice groups, which were not assessed at the study baseline before the antibiotics were introduced. Moreover, the administration of antibiotics to the GI microbiota dysbiosis group may have directly modulated inflammatory responses or the reciprocal case, irrespective of ovarian cancer. Although the role of microbiota compositional changes in the pathways that regulate cellular proliferation/apoptosis markers in carcinogenesis is not fully understood, we did not perform immunostaining for proliferation and apoptosis markers in this study. While mouse models are useful in understanding mechanistic patterns, human and mouse microbiota are intrinsically unique [63]. The study used a general mouse model of EOC that did not differentiate between histologic subtypes. Further research is needed to compare GI and ovarian tumor microbial characteristics and metabolic function. Therefore, future prospective studies should incorporate the high-grade serous ovarian carcinoma EOC subtype using preclinical mouse models and patient recruitment.

## 5. Conclusions

This study detected unique bacteria in GI dysbiosis that were linked to systemic inflammatory responses during ovarian cancer. However, it is unknown if these bacteria favor the occurrence and development of ovarian tumor. Malignant ovarian cancer cells are present in ascites and peritoneal washings during early disease, and tumor-associated peritoneal bacteria may become clinically relevant if they can also be detected in the fecal samples of high-risk patients. As ovarian cancer screening methods are absent in routine practice, bacteria-based candidates could serve as biomarkers for the development of clinical tests for early disease detection.

## Figures and Tables

**Table 1:** Key bacteria and clinical associations.

Taxonomy	Inflammatory Marker Association in Study Mice	Clinical Relevance
**Genus**		
Parabacteroides	Positive correlation with CRP serum levels with combined groups.	Increased tumor tissue abundance compared to colorectal samples in noncancer patients [43] and adjacent normal tissue [44] in patients with colorectal cancer.
Roseburia	Negative correlation with CRP serum levels with combined groups.	Increased fecal abundance in patients with cervical cancer [45]; reduced fecal abundance in patients with colorectal cancer [46,47].
Emergencia	Negative correlation with CRP serum levels with combined groups.	Mediates pathway of carnitine converted trimethylamine N-oxide accumulation involved in cardiovascular disease susceptibility [48].
**Species**		
Ruminococcus faecis	Negative correlation with CRP serum levels with combined groups.	Increased fecal abundance in patients with colorectal cancer and obesity compared to control group [49]; differential expression from rectal and ileum tissue samples predicted presence or absence of upper GI tract involvement in patients with Crohn’s disease [50].
Parabacteroides distasonis	Positive correlation with CRP serum levels with combined groups.	Increased fecal abundance in patients with Lynch syndrome [51]; decreased fecal abundance in patients with multiple sclerosis [52]. Cultured from isolated microlesions of the gut wall in Crohn’s disease [53].
Roseburia faecis	Negative correlation with CRP serum levels with combined groups.	Included in a fecal microbial biosignature-based predictive model differentiating between individuals with and without early-stage lung cancer [54].
Emergencia timonensis	Negative correlation with CRP serum levels with combined groups.	Mediates pathway of carnitine converted trimethylamine N-oxide accumulation involved in cardiovascular disease susceptibility [55].

## Data Availability

Not applicable.

## Supplementary Materials

The following supporting information can be downloaded at: https://www.mdpi.com/article/10.3390/cancers14123022/s1, Supplementary data, Spearman’s correlation analysis of inflammatory marker serum levels and bacterial abundance using average values over 3, 6, and 10 weeks, Table S1: CRP and Phylum, Table S2: CRP and Genus, Table S3: CRP and Species, Table S4: IL-6 and Phylum, Table S5: IL-6 and Genus, Table S6: IL-6 and Species, Table S7: TNF-α and Phylum, Table S8: TNF-α and Genus, and Table S9: TNF-α and Species.
